# Long Noncoding RNAs in the Prediction of Survival of Patients with Digestive Cancers

**DOI:** 10.5152/tjg.2022.22017

**Published:** 2023-01-01

**Authors:** Shu Zhao, Peng Li, Gang Zhou

**Affiliations:** 1 Department of Medical Oncology, The Second Medical Center and National Clinical Research Center for Geriatric Diseases, Chinese PLA General Hospital, Beijing, China

**Keywords:** Digestive cancers, incRNAs, prognosis

## Abstract

**Background:**

Long noncoding RNAs have been known to be involved in various cancers. This study aimed to find a long noncoding RNA signature to predict the prognostic risk of patients with digestive cancers, including esophageal carcinoma, stomach adenocarcinoma, liver hepatocellular carcinoma, and pancreatic adenocarcinoma.

**Methods:**

After screening differentially expressed long noncoding RNAs in 4 digestive cancers from The Cancer Genome Atlas database, the prognostic significance of the above differentially expressed long noncoding RNAs was evaluated by Kaplan–Meier analysis. Target genes of the corresponding differentially expressed long noncoding RNAs were predicted by StarBase. We performed bioinformatics methods, including gene ontology annotation and Kyoto Encyclopedia of Genes and Genomes pathway enrichment analysis, to explore the role and molecular mechanisms of differentially expressed long noncoding RNAs and predicted target genes in tumor progression.

**Results:**

A total of 4 differentially expressed long noncoding RNAs (AC093895.1, CASC9, LINC01980, and HOXC-AS2) with a significant prognostic value were identified. Moreover, 6 target genes were obtained. Also, functional enrichment analysis showed that these 4 DELs were mainly related to the regulation of mRNA metabolic process, regulation of RNA stability, mRNA binding, RNA localization, and spliceosome.

**Conclusion:**

The prognostic differentially expressed long noncoding RNAs and target genes in the digestive cancers were obtained, which may provide a novel direction for treatment and prognosis improvement of digestive cancers.

**Main Points**

- Four differentially expressed long noncoding RNAs (DELs; AC093895.1, CASC9, HOXC-AS2, and LINC01980), which are potential and vital prognostic biomarkers, were identified in digestive cancers (DCs) based on The Cancer Genome Atlas database. Our study is the first one to suggest that AC093895.1 may exert oncogene roles in DCs.
- A total of 6 hub target genes (DKC1, DGCR8, IGF2BP2, RBFOX2, FBL, and UPF1) were identified for the function analyses.
- These 4 DELs can regulate variant genes and signaling pathways in DCs. From the perspective of pan-cancer, our study may lay the molecular foundation and bring the prognostic lncRNAs or target genes to clinics in the future.

## Introduction

Digestive cancers (DCs) are leading causes of cancer-related deaths worldwide and are also common malignancies with high morbidity and mortality in China,^1,2^ including esophageal carcinoma (ESCA), stomach adenocarcinoma (STAD), rectal adenocarcinoma (READ), liver hepatocellular carcinoma (LIHC), and pancreatic adenocarcinoma (PAAD). As main malignancies of the digestive tract, ESCA, STAD, and colorectal cancer (CRC) are ranked in the top 10 for morbidity of tumors with incidences of 5.3%, 8.2%, and 9.0% respectively.^3^ As most of DCs are diagnosed at an advanced stage, which delays optimal therapy, early and timely diagnosis remains important for patients, and novel biomarkers are necessary for early-stage monitoring of the DCs.^4^ Our study aimed to identify potential early diagnostic biomarkers and explore their molecular mechanisms in DCs.

Long noncoding RNAs (lncRNAs) are pervasive transcripts of more than 200 nucleotides in length.^5^ Recently, an increasing number of lncRNAs have been demonstrated to be vital biomarkers for clinical diagnosis and prognosis.^6,7^ Furthermore, lncRNAs can exert a crucial role in tumor progression, metastasis, and recurrence via the regulation of various biological processes.^8-11^ Additionally, lncRNAs are also identified to be associated with the diagnosis and prognosis of STAD,^12,13^ ESCA,^14^ LIHC,^15^ and PAAD.^16^ Therefore, we aimed to elucidate the functions and prognostic significance of lncRNAs in DCs.

In this study, lncRNA expression profiles related to ESCA, STAD, LIHC, and PAAD were downloaded from The Cancer Genome Atlas (TCGA) database. After identifying differentially expressed lncRNAs (DELs), correlations between these DELs and survival were examined. Furthermore, we explored the molecular mechanisms of DELs in DCs through bioinformatics methods, including gene ontology (GO) annotation and Kyoto Encyclopedia of Genes and Genomes (KEGG) pathway enrichment analysis.

## Materials and Methods

### Data Acquisition and Processing

Gene expression data and clinical data were downloaded from the TCGA database (https://tcga-data.nci.nih.gov/tcga/), including ESCA, STAD, LIHC, and PAAD tissue samples as well as corresponding normal tissue samples.

### Identification of Differentially Expressed Long Noncoding RNAs

The edge R package was used to filter the DELs with |logFC| > 1 and adjusted *P* < .05. Then, we screened the DELs from the above with |logFC| > 2 and adjusted *P* < .01, followed by the DELs intersection in the DCs. Intersection was performed on the obtained DELs and their prognostic value was demonstrated by Kaplan–Meier analysis.

### Prediction of the Target Genes of Differentially Expressed Long Noncoding RNAs

StarBase (http://starbase.sysu.edu.cn/) was used to predict the potential target genes, which can be used to perform the miRNA interaction and protein interaction analysis of lncRNAs.

### Gene Ontology Annotation and Kyoto Encyclopedia of Genes and Genomes Pathway Analysis

Gene Ontology (GO) and Kyoto Encyclopedia of Genes and Genomes (KEGG) enrichment of target genes associated with lncRNAs were analyzed by Metascape (http://metascape.org).^17^ Restrictions: *P* < .01 and a minimum count of 3 and enrichment factor > 1.5 were identified as statistical significance.

## The Cellular Localization of Long Noncoding RNAs

LncLocator (https://LncLocatorwww.csbio.sjtu.edu.cn/bioinf/lncLocator/) was used to detect the subcellular localization of lncRNAs based on the lncRNA gene sequences from UCSC (https://genome-asia.ucsc.eduk/index.html).

### Statistical Analysis

R software was used for all the statistical analyses involving differential expression, Cox regression analysis, and Kaplan–Meier curves. Statistical significance was defined as *P* < .05.

## Results

### Differentially Expressed Long Noncoding RNAs in Digestive Cancers

**Figure 1.**
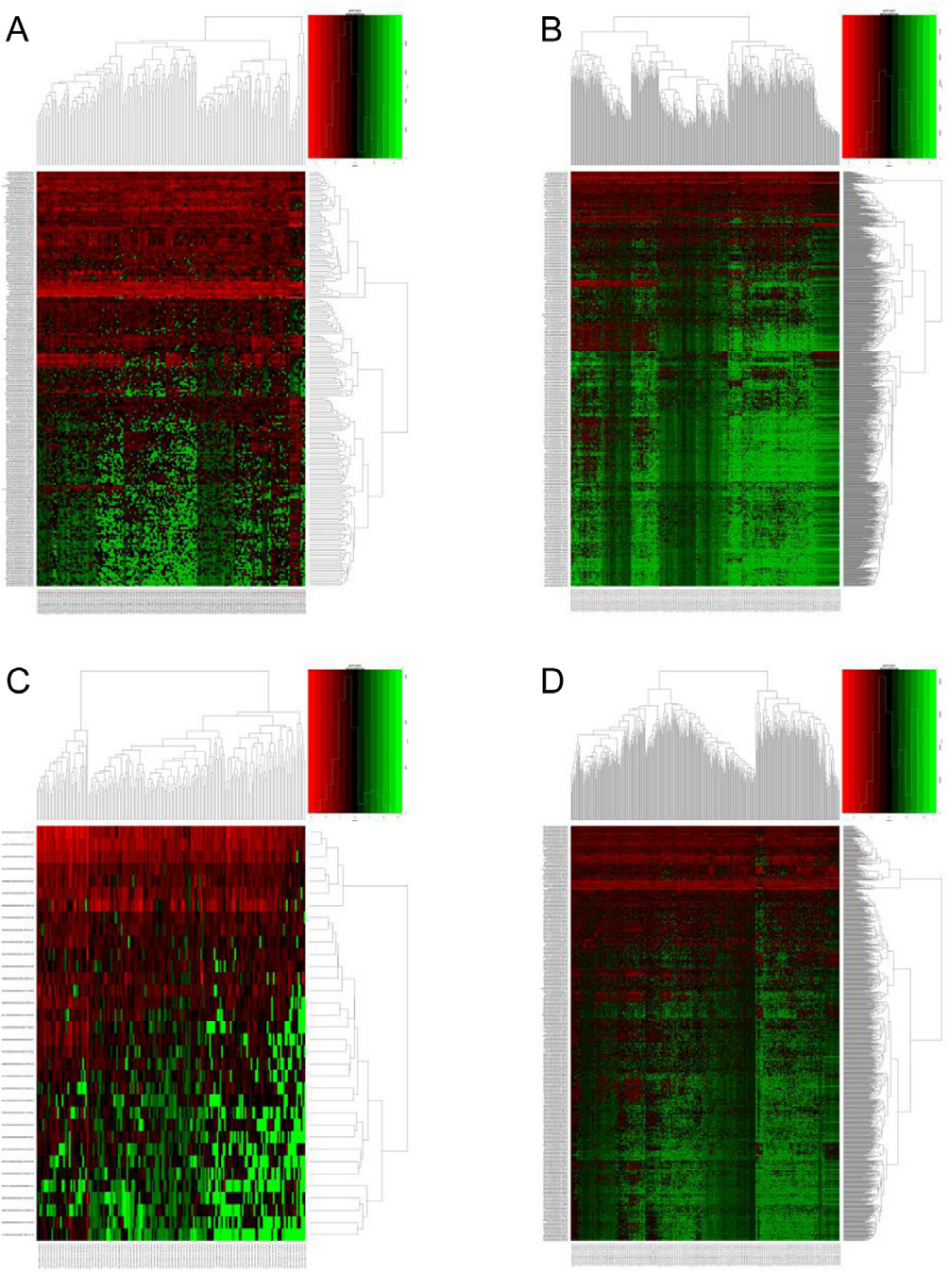
Heat map of differential expressed lncRNAs in 4 digestive cancers. (A) Esophageal carcinoma; (B) liver hepatocellular carcinoma; (C) pancreatic adenocarcinoma; (D) stomach adenocarcinoma.

**Figure 2.**
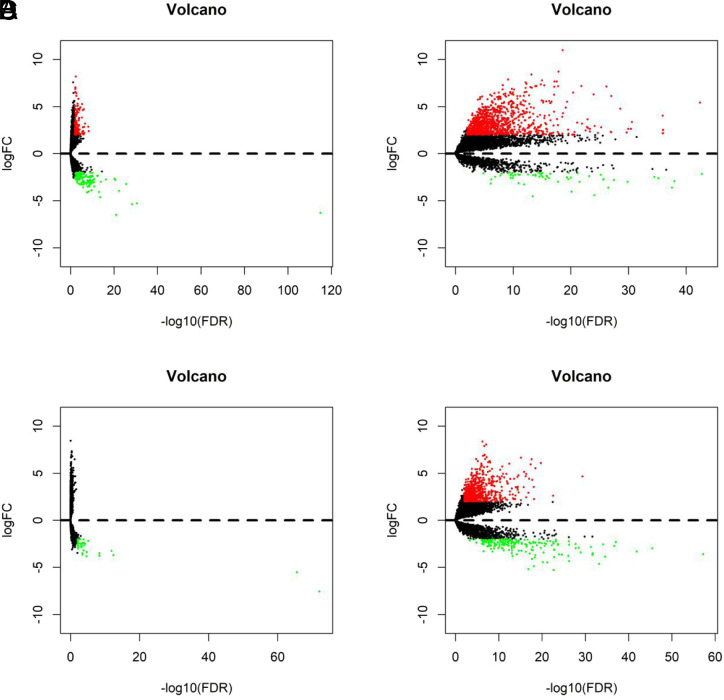
Volcano map of differential expressed lncRNAs in 4 digestive cancers. (A) Esophageal carcinoma; (B) liver hepatocellular carcinoma; (C) pancreatic adenocarcinoma; (D) stomach adenocarcinoma. The volcano map was drawn by the R language gplots package. The difference in expression of lncRNAs (logFC > 2, *P* < .01) was marked in red, and the difference in expression of downregulated lncRNAs (logFC > −2, *P* < .01) was marked in green.

After collecting lncRNA expression data of ESCA (160 tumor and 11 normal samples), STAD (375 tumor and 32 normal samples), LIHC (374 tumor and 50 normal samples), and PAAD (178 tumor and 4 normal samples), R language edge R package was used for statistical analysis and screening (|logFC| > 2, *P* < .01) (Figures 1 and 2).

### Prognostic Values of Dysregulated Long Noncoding RNAs

**Figure 3.**
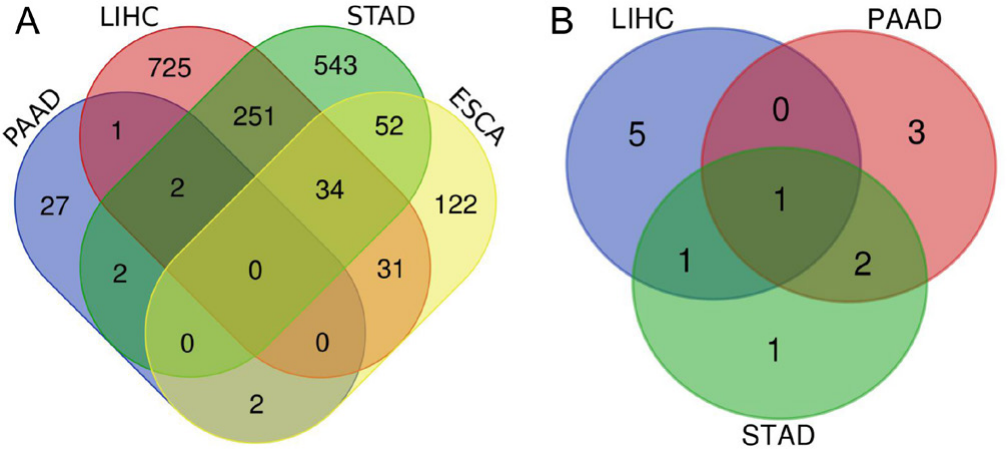
Venn map of differential expressed long noncoding RNAs (lncRNAs) associated with the prognosis of digestive cancers. (A) Total differential expressed lncRNAs; (B) prognostic differential expressed lncRNAs. LIHC, liver hepatocellular carcinoma; STAD, stomach adenocarcinoma PAAD, pancreatic adenocarcinoma.

**Figure 4.**
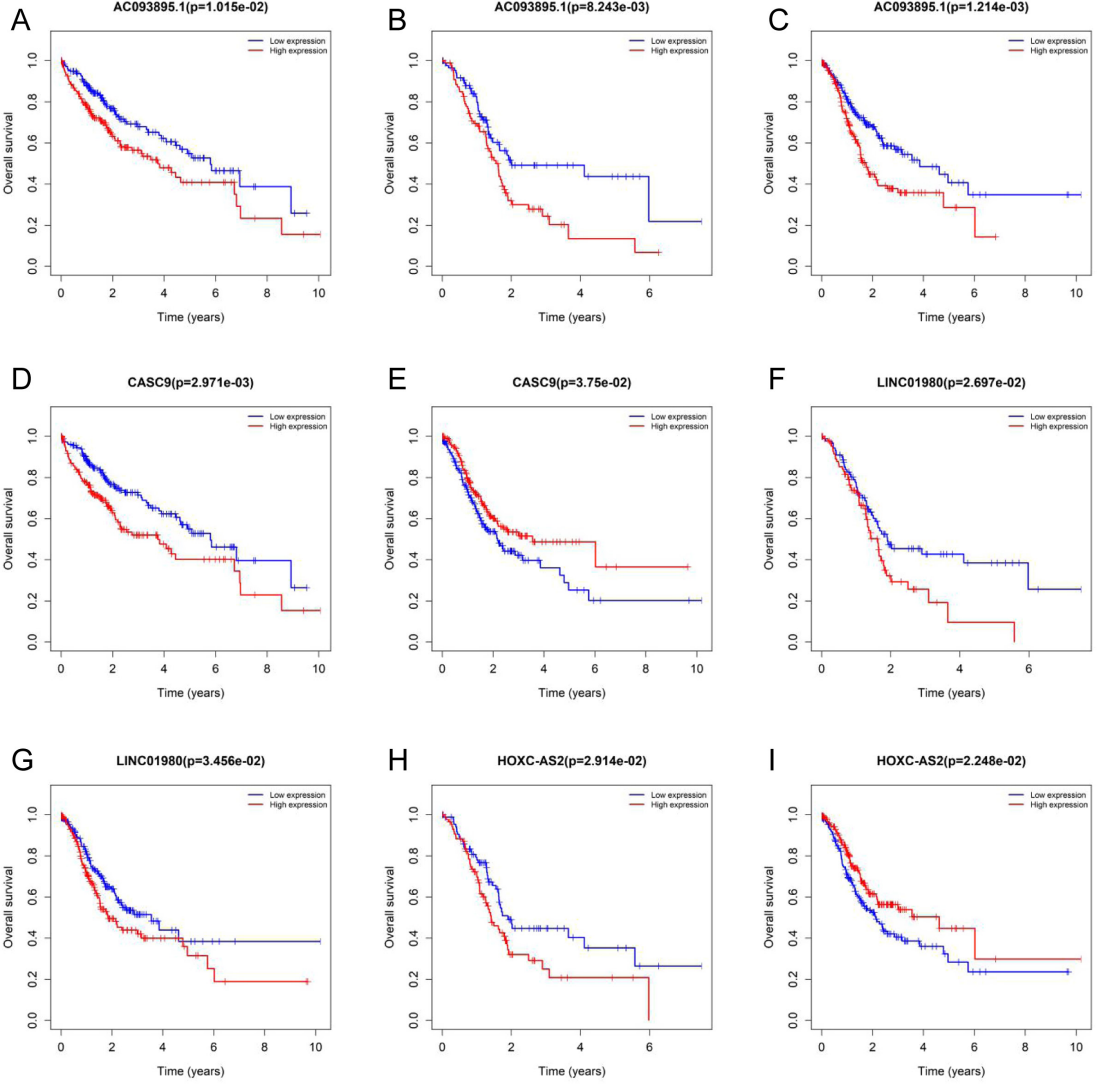
Kaplan–Meier survival curve of 4 differential expressed long noncoding RNAs in digestive cancers. (A) LIHC AC093895.1; (B) PAAD AC093895.1; (C) STAD AC093895.1; (D) LIHC CASC9; (E) STAD CASC9; (F) PAAD LINC01980; (G) STAD LINC01980; (H) PAAD HOXC-AS2; (I) STAD HOXC-AS2. The blue color represents the low expression of the patient, and the red color represents the high expression of the patient. LIHC, liver hepatocellular carcinoma; STAD, stomach adenocarcinoma PAAD, pancreatic adenocarcinoma.

By the intersection of candidate lncRNAs, a total of 36 DELs were obtained in 3 cancers (Figure 3A). We further identified the above DELs based on survival time and removed the DELs with no significant values. After intersection, AC093895.1 was found in LIHC/PAAD/STAD intersection, CASC9 in LIHC/STAD intersection, and LINC01980 and HOXC-AS2 in PAAD/STAD intersection (Figure 3B). We further explored the prognostic values of these 4 DELs in DCs (Figure 4). In LIHC, PAAD, and STAD, high expression of AC093895.1 had a lower survival rate, which suggested that AC093895.1 was a risk factor. Interestingly, the high expression of CASC9 had a worse prognosis than the low one in LIHC while the high expression of CASC9 had a better prognosis in STAD, suggesting that CASC9 was a risk factor in LIHC and a protective factor in STAD. Similarly, the high expression of HOXC-AS2 in STAD had a high survival rate while a poor survival rate in PAAD. High LINC01980 expression was related to a poor prognosis in PAAD and STAD, indicating that LINC01980 was a risk factor in PAAD and STAD.

### Prediction of the Target Genes of Differentially Expressed Long Noncoding RNAs

**Figure 5.**
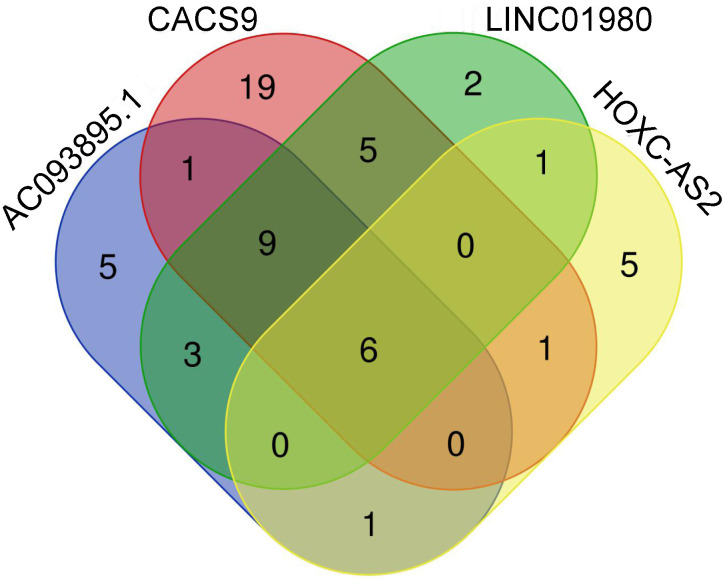
Venn map of the common target genes of 4 differential expressed long noncoding RNAs in digestive cancers.

We further explored the binding proteins of AC093895.1, CASC9, LINC01980, and HOXC-AS2. 25, 41, 26, and 14 target genes were identified to be associated with these four lncRNAs, respectively. Moreover, 6 targets (DKC1, DGCR8, IGF2BP2, RBFOX2, FBL, and UPF1) were common in these 4 lncRNAs (Figure 5).

### Functional Enrichment Analysis of Long Noncoding RNA Targets

**Figure 6.**
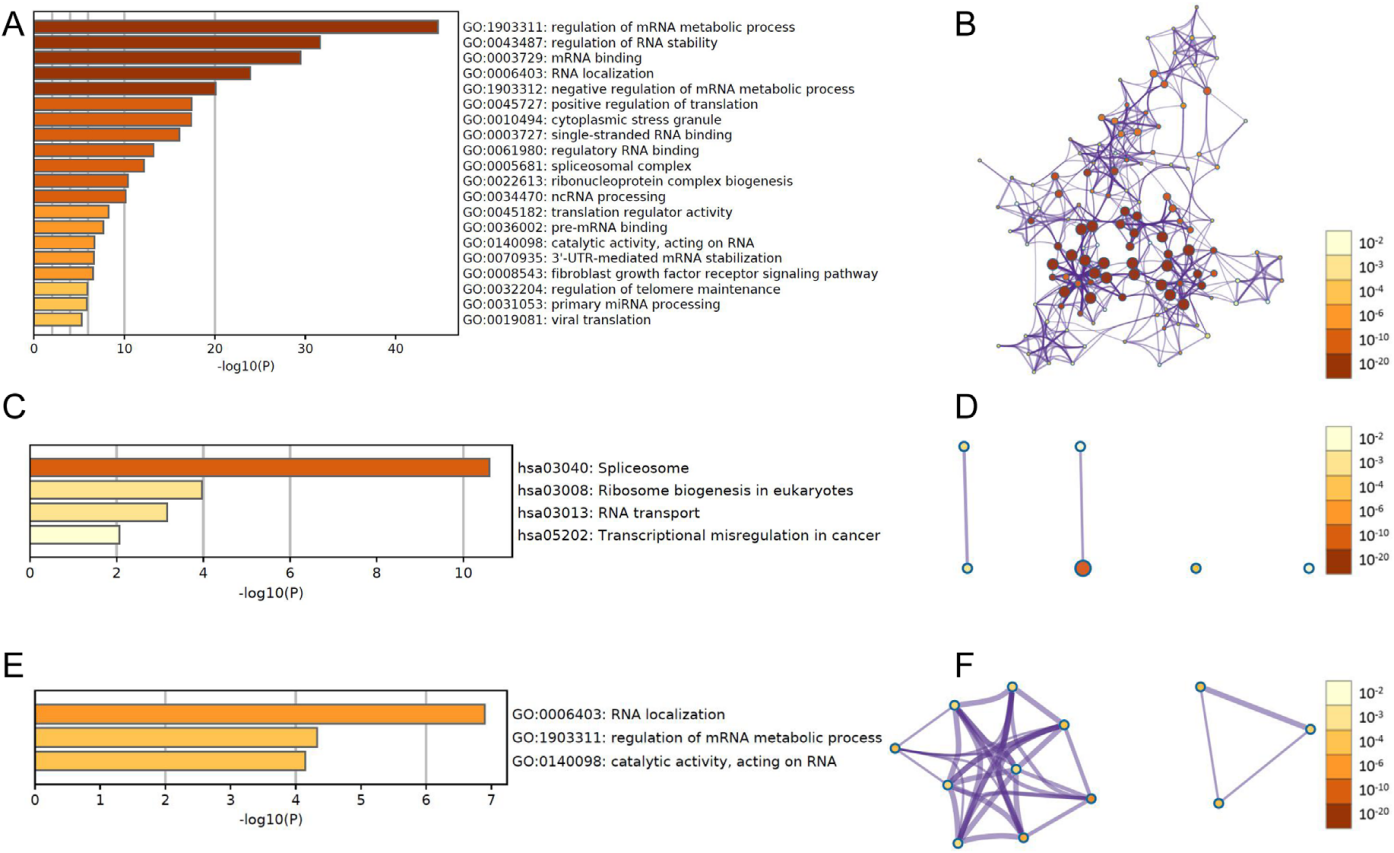
Significant enrichment analysis of gene ontology (GO) and Kyoto Encyclopedia of Genes and Genomes (KEGG) pathways of target genes. (A-B) Gene ontology in all target genes; (C-D) KEGG in all target genes; (E-F) GO in 6 common target genes.

We performed GO and KEGG enrichment analysis to explore the roles of AC093895.1, CASC9, LINC01980, and HOXC-AS2 in the malignant progress of DCs. The results of GO and KEGG indicated that these targeted genes were mainly related to the regulation of mRNA metabolic process and RNA stability, mRNA binding, and RNA localization via spliceosome (Figure 6A-D). Furthermore, the 6 common targets were mainly involved in RNA localization, regulation of mRNA metabolic process, and catalytic activity, acting on RNA (Figure 6E, F).

### The Cellular Location

**Figure 7.**
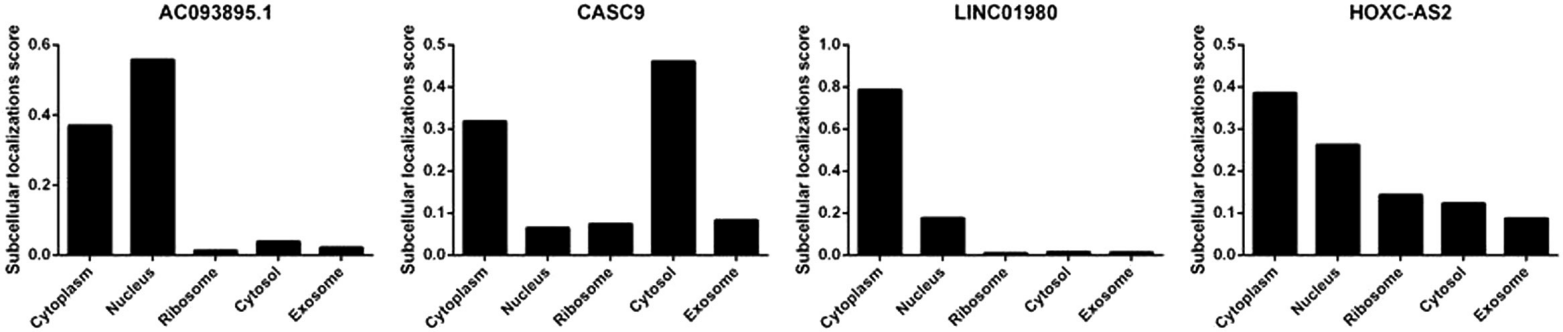
The cellular location of 4 differential expressed long noncoding RNAs in digestive cancers.

The cellular localizations of 4 overlapping lncRNAs were explored due to the vital molecular mechanisms. The results indicated that AC093895.1 was mainly in the nucleus and cytoplasm (score: 0.56 and 0.37, respectively), CASC9 mainly in cytosol and cytoplasm (score: 0.46 and 0.32, respectively), LINC01980 mainly in the cytoplasm (score: 0.79), and HOXC-AS2 mainly in cytoplasm and nucleus (score: 0.39 and 0.26, respectively) (Figure 7).

## Discussions

The prognosis DCs is relatively poor at present. Recently, lncRNAs have been suggested to serve as potential diagnostic biomarkers for DCs and play a critical role in various biological behaviors, including tumor proliferation, invasion, and metastasis.^8,18^ Despite the increasing number of studies on the perspective of pan-cancer, the prognostic significance of lncRNAs in DCs was rarely explored.^19,20^ Therefore, it is clinically relevant to explore the roles and potential mechanisms of lncRNAs in DCs. This study aimed to identify lncRNA markers associated with the prognosis of 4 DCs and explore the potential functions and mechanisms. In our study, 36 DELs in DCs were identified. We further confirmed that AC093895.1, CASC9, HOXC-AS2, and LINC01980 were vital prognostic biomarkers in 4 DCs.

The high expression of AC093895.1 in LIHC, PAAD, and STAD was associated with a poor survival rate, suggesting that AC093895.1 was a risk factor. To the best of our knowledge, this study is the first one to suggest that AC093895.1 may exert oncogene roles in DCs. A further study is warranted to explore the functions of AC093895.1 in cancers. A previous study has demonstrated that low CASC9 expression in hepatocellular carcinoma (HCC) had a better prognosis,^15^ which was consistent with our results in LIHC. Besides, high CASC9 expression exerted a tumor suppressor role in gastric cancer (GC) and positively correlated with lymph node metastasis and TNM stage,^21^ which was also similar in STAD in our study. Moreover, CASC9 serves as an oncogene in multiple tumors, including CRC,^22^ bladder cancer,^23^ and papillary thyroid cancer.^24^ The relationship between HOXC-AS2 and digestive cancers remains unclear. However, HOXC-AS2 was elevated and HOXC-AS2 knockdown suppressed the invasion, migration, and EMT process in glioma and non-small cell lung cancer.^25,26^ Furthermore, high HOXC-AS2 expression in STAD had a high survival rate while a poor survival rate in PAAD, indicating that HOXC-AS2 may exert different effects in various cancer types. In our study, we identified LINC01980 as a risk factor in PAAD and STAD. The specific role of LINC01980 is rarely reported in tumor biology. LINC01980 can play an oncogenic role in esophageal squamous cell carcinoma.^27,28^ Upregulation of LINC01980 related to a low survival rate in HCC.^29^ LINC01980 was elevated in LIHC, suggesting that LINC01980 may serve as an oncogene in LIHC. AC093895.1, CASC9, HOXC-AS2, and LINC01980 may be involved in tumor progression, and the molecular mechanism remains to be further verified.

Increasing evidence has demonstrated that the effects and mechanisms of lncRNAs on the initiation and development may be through network regulation and signaling pathways.^30,31^ Thus, we further identified the target genes that were involved in the biological function and pathways. Targeted genes were mainly involved in the regulation of mRNA metabolic process and RNA stability, mRNA binding, and RNA localization. Previous studies have shown that lncRNAs, partly in the cytoplasm and cytosol, could regulate the mRNA stability, translation, and post-transcriptional modification, as well as cell signal transduction.^32^ Additionally, we found 6 common targets of AC093895.1, CASC9, HOXC-AS2, and LINC01980. DKC1 was reported to promote CRC angiogenesis and metastasis via HIF-1α transcription.^33^ DGCR8 can promote tumor cell migration and invasion through targeting TGF-β in triple-negative breast cancer and can act as the target gene of other lncRNAs.^34,35^ IGF2BP2 was repressed by lncRNA 91H to promote CRC tumorigenesis and was interacted by GHET1 through regulating the AKT/mTOR and Wnt/β-catenin pathways.^36,37^ LncRNA MALAT1 promotes ovarian cancer progression via regulating RBFOX2-mediated alternative splicing.^38^ Fibrillarin (FBL) is an essential nucleolar protein that takes part in pre-rRNA methylation and processing.^39^ UPF1 was demonstrated to exert critical roles in various cancers, including lung adenocarcinoma,^40^ HCC,^41,42^ and CRC.^43^ Therefore, we speculated that AC093895.1, CASC9, HOXC-AS2, and LINC01980 could affect initiation, progression, and biological behaviors in DCs via the regulation of the above tumor-related target genes and pathways.

## Conclusions

In summary, we identified 4 DELs (AC093895.1, CASC9, HOXC-AS2, and LINC01980), which are potential and vital prognostic biomarkers in DCs based on the TCGA database. Moreover, 6 hub-target genes were also identified for the function analyses. The results indicated that these 4 DELs can regulate variant genes and signaling pathways in DCs. However, both in vitro and in vivo experiments are needed for further functional analysis of these DELs and their target genes. From the perspective of pan-cancer, this study may lay the molecular foundation and bring the prognostic lncRNAs and target genes to clinics in the future.
